# Amplicon sequencing based profiling of bacterial diversity from Krossfjorden, Arctic

**DOI:** 10.1016/j.dib.2018.11.101

**Published:** 2018-11-27

**Authors:** Bhavya Kachiprath, Jayesh Puthumana, Jayanath Gopi, Solly Solomon, K.P. Krishnan, Rosamma Philip

**Affiliations:** a Department of Marine Biology, Microbiology and Biochemistry, School of Marine Sciences, Cochin University of Science and Technology, Kochi 682016, Kerala, India; b National Centre for Aquatic Animal Health, Cochin University of Science and Technology, Fine Arts Avenue, Kochi 682016, Kerala, India; c Fishery Survey of India, Mormugao Zonal Base, Vasco da Gama, Goa, India; d National Centre for Polar and Ocean Research, Ministry of Earth Sciences, Government of India, Goa, India

**Keywords:** Arctic, Bacterial diversity, Metagenome, Amplicon sequencing, Illumina

## Abstract

In this study, Illumina Miseq sequencing of 16S rRNA gene amplicon was performed on sediments collected from Krossfjorden, Arctic for analyzing the bacterial community structure. Metagenome contained 15,936 sequences with 5,809,491 bp size and 53% G+C content. Metagenome sequence information are now available at NCBI under the Sequence Read Archive (SRA) database with accession no. SRP159159. Taxonomic hits distribution from MG-RAST analysis revealed the dominance of Alpha- and Gamma-subdivisions of Proteobacteria (88.89%) along with Bacteriodetes (8.89%) and Firmicutes (2.22%). Predominant species were *Alteromonadales bacterium* TW-7 (24%), *Pseudoalteromonas haloplanktis* (20%) and *Pseudoalteromonas* spp. SM9913 (18%). MG-RAST assisted analysis also detected the presence of a variety of marine taxa like *Bacteriodes, Pseudovibrio, Marinobacter, Idiomarina, Teredinibacter,* etc. which take part in key ecological functions and biogeochemical activities of Arctic fjord ecosystems.

## Specifications table

**Table:** 

Subject area	*Biology*
More specific subject area	*Polar metagenomics*
Type of data	*FastaQ file*
How data was acquired	*NGS sequencing on Illumina MiSeq platform*
Data format	*Raw data*
Experimental factors	*Fjord sediment samples from Krossfjorden, Arctic*
Experimental features	*Metagenomic DNA extraction from Krossfjorden sediment, NGS sequencing on Illumina MiSeq platform and MG-RAST analysis of NGS data*
Data source location	*Krossfjorden, Arctic (79°08׳60"N, 11°44׳59"E)*
Data accessibility	*The data of this metagenome is available in the NCBI BioSample Submission Portal as Bioproject ID:* PRJNA488527 *and SRA accession no.:*SRP159159. https://www.ncbi.nlm.nih.gov/sra/SRP159159.

## Value of the data

• Present study reveals the bacterial community structure in Arctic fjords, a least explored extreme environment undergoing rapid changes due to climate variability.
• Metagenome based study enabled the detection of Arctic bacterial community including unculturable bacterial population.
• Dominance of gram negative bacteria (97.78%) in the Krossfjorden sediments limiting the gram positive to a significantly low level 2.22% was an important observation.
• Proteobacteria dominated the bacterial community followed by Bacteriodetes and Firmicutes.

## 1 Direct link to deposited data

Deposited data can be found here: https://www.ncbi.nlm.nih.gov/sra/?term=SRP159159.

## 2 Data

During recent years, metagenome based approaches has helped in unraveling the microbiome diversity in various eco-habitats overcoming the shortfalls of culture based methods, since majority of the microbes are unculturable and thereby go undetected in conventional methods. Supported with Next Generation Sequencing Technology, Metagenomics can provide vast information about the enormous uncultured microbial populations existing in any environment [1]. Microbial communities inhabiting extreme environments are adapted to extreme conditions thereby serving as a repository of novel genes and bioactive molecules. Collective operation of metagenomics, NGS platforms and annotation tools elicit the exploration of extremophiles from diverse environments [2], [3]. Present study is focused on the taxonomic composition of bacterial assemblages in Krossfjorden sediments.

**Fig. 1:**
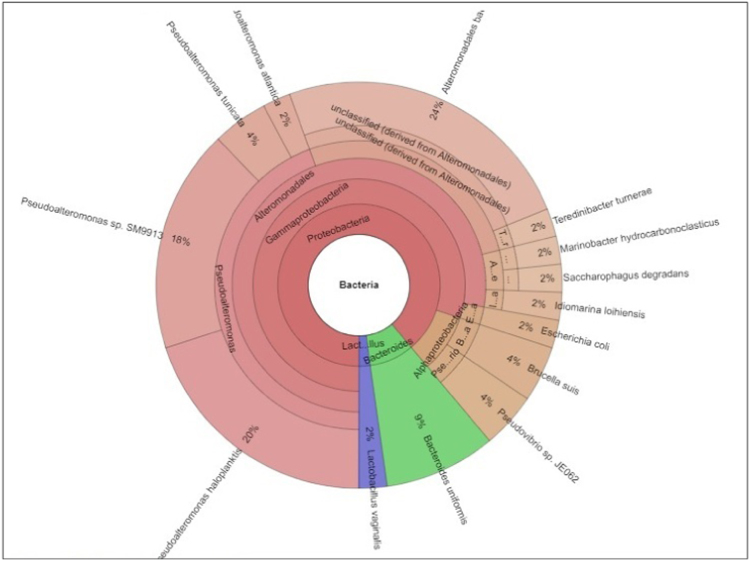
Interactive Krona chart for visualization of bacterial communities detected from Krossfjorden sediments.

A total of 15,796 reads were analyzed and 2907 operational taxonomic units (OTU) were identified in order to reveal the diversity of bacterial flora. All the resulting fragments or OTUs were then classified into 3 phyla, 4 classes, 6 orders, 9 families and 11 genera. MG-RAST analysis for phylum level classification revealed the preponderance of Proteobacteria (88.89%) followed by Bacteriodetes (8.89%) and Firmicutes (2.22%). The classified OTUs represent distinct orders dominated by Alteromonadales, Bacteriodales, Rhizobiales and Rhodobacteriales. Family level dominance was shown by Pseudoalteromonadaceae (40.44%), Bacteriodaceae (8.89%), Rhodobacteriaceae (4.44%), Alteromonadaceae (4.44%) and Brucellaceae (4.44%). Nevertheless, an unclassified bacterial group derived from Alteromonadales (26.67%) was also found among the Krossfjorden bacterial families. The analysis identified 11 genera among which the most dominant was *Pseudoalteromonas* (44.44%) followed by *Bacteriodes* (8.89%), *Pseudovibrio* (4.44%) and *Brucella* (4.44%). Species level identification showed that *Alteromonadales bacterium* TW-7 (24%), *Pseudoalteromonas haloplanktis* (20%) and *Pseudoalteromonas* sp. SM9913 (18%) were the predominant ones (Fig. 1) [4].

## 3 Experimental design, materials and methods

In this study, sediment samples were collected from Krossfjorden (79°08׳60"N, 11°44׳59"E) using a Van Veen Grab during Indian Scientific Expedition to the Arctic (2015–2016) during summer. Sediment samples were suspended in sterilized virus free seawater and subjected to low speed (320 × *g*) centrifugation for the removal of coarse sediment particles followed by high speed (9000 × *g*) centrifugation for collecting the fine sediment fraction with bacteria. The supernatant was subjected to sequential filtration using 0.8 μ, 0.45 μ and 0.22 μ filter membranes for the separation of bacterial fraction [5]. Fine sediment obtained after high speed centrifugation and the residue on filter membranes were used for metagenomic DNA extraction as per modified Zhou et al. [6] protocol. Next Generation Sequencing was done using Illumina Miseq at Interpretomics, Bangalore. Quality and quantity of extracted DNA were analysed by NanoDrop ND-2000 and Qubit. The 16S rRNA libraries were prepared from the QC passed DNA sample using 16S rRNA gene universal primers (Forward Primer: ACTCCTACGGGAGGCAGCAG and Reverse Primer: GGACTACHVGGGTWTCTAAT) with standard Illumina barcodes and adapters. The Amplicons were further purified using Ampure XP beads. The barcoded libraries were validated by Agilent DNA 1000 Bioanalyser and quantified using Qubit DNA BR reagent assay. The quantified libraries were pooled and sequenced using MiSeq.

Raw sequences from Illumina Miseq were processed and analysed using Metagenomic Rast Server (MG-RAST) version 4.0.3 (http://metagenomics.anl.gov/). Raw data were uploaded as FASTAQ files after demultiplexing of paired-end reads. Reads generated after quality processing and deduplication by MG-RAST pipeline analysis were subjected to taxonomic analysis. MG-RAST pipeline has provided an estimate of bacterial abundances present in Krossfjorden sediments and based on these, an assessment was carried out to evaluate the bacterial diversity within the sample.

### 3.1 Nucleotide sequence accession number

Metagenome sequence data from this study were submitted to the NCBI Sequence Read Archive (SRA) under the accession number: SRP159159.
